# Enhanced Neointima Formation Following Arterial Injury in Immune Deficient Rag-1−/− Mice Is Attenuated by Adoptive Transfer of CD8^+^ T cells

**DOI:** 10.1371/journal.pone.0020214

**Published:** 2011-05-24

**Authors:** Paul C. Dimayuga, Kuang-Yuh Chyu, Jonathan Kirzner, Juliana Yano, Xiaoning Zhao, Jianchang Zhou, Prediman K. Shah, Bojan Cercek

**Affiliations:** Division of Cardiology, Oppenheimer Atherosclerosis Research Center, Cedars-Sinai Heart Institute, Los Angeles, California, United States of America; Universität Würzburg, Germany

## Abstract

T cells modulate neointima formation after arterial injury but the specific T cell population that is activated in response to arterial injury remains unknown. The objective of the study was to identify the T cell populations that are activated and modulate neointimal thickening after arterial injury in mice. Arterial injury in wild type C57Bl6 mice resulted in T cell activation characterized by increased CD4^+^CD44^hi^ and CD8^+^CD44^hi^ T cells in the lymph nodes and spleens. Splenic CD8^+^CD25^+^ T cells and CD8^+^CD28^+^ T cells, but not CD4^+^CD25^+^ and CD4^+^CD28^+^ T cells, were also significantly increased. Adoptive cell transfer of CD4^+^ or CD8^+^ T cells from donor CD8−/− or CD4−/− mice, respectively, to immune-deficient Rag-1−/− mice was performed to determine the T cell subtype that inhibits neointima formation after arterial injury. Rag-1−/− mice that received CD8^+^ T cells had significantly reduced neointima formation compared with Rag-1−/− mice without cell transfer. CD4^+^ T cell transfer did not reduce neointima formation. CD8^+^ T cells from CD4−/− mice had cytotoxic activity against syngeneic smooth muscle cells in vitro. The study shows that although both CD8^+^ T cells and CD4^+^ T cells are activated in response to arterial injury, adoptive cell transfer identifies CD8^+^ T cells as the specific and selective cell type involved in inhibiting neointima formation.

## Introduction

Clinical evidence suggest that the T cell immune response is involved in restenosis, the process of re-narrowing of the artery after percutaneous coronary intervention (PCI), but the specific T cell subtypes involved remain to be elucidated [1]–[3]. Current understanding of immune function in the vascular wall is based mostly on alloreactive responses, but little is known about syngeneic T cell responses, which is presumably what would happen in the immune response to arterial injury. This is a significant issue considering that options to treat restenosis include the use of immune-suppressing drugs [4]–[6]. In addition, there is the possibility of persistent immune activation after PCI [7].

Specific immune activation signals after arterial injury remain undefined but sources of non-antigen specific signals include release of intracellular material such as uric acid by injured cells [8], or adjuvant-like activity by heat shock proteins [9]. In addition, lipid neoantigens produced after arterial injury may be important signaling molecules [10].

Neointimal thickening is the underlying mechanism that drives restenosis and recent experimental reports suggest that T cell recruitment into the arterial wall promotes the process [11]–[13]. On the other hand, experimental studies have also demonstrated that neointima formation is significantly augmented in immune-compromised animals, specifically those with T cell deficiency [14]–[18]. We have previously reported that adoptive transfer of T cells into immune-deficient Rag-1−/− mice reduced neointima formation [16]. Common to all these reports is the involvement of T cells in neointima formation. However, the T cell response to arterial injury is not well characterized and its kinetics undefined. Natural killer (NK) T cells augment neointima formation [10] but it remains unclear if other subsets of T cells play defined roles in the response [6]. It is thus important to identify which T cells are involved in modulating the response to vascular injury.

We provide evidence that arterial cuff injury results in T cell immune activation, characterized by a robust CD8^+^ T cell response. To help elucidate the T cell subset(s) involved in neointima formation after vascular injury, we used adoptive transfer of CD4^+^ or CD8^+^ T cells to immune-deficient Rag-1−/− mice [19]. The adoptive transfer model allowed for the direct study of the role of distinct subsets of immune cells. We have reported that B cells and immunoglobulin reduce neointima formation after arterial injury [15], [20]. Thus, to test the role of specific T cell subsets, adoptive transfer of specific T cell subtypes provided the best approach to exclude the effect of B cells. The results show that CD8^+^ T cells are likely the subtype involved in inhibiting neointima formation.

## Results

### Characterization of T cell response to arterial injury in WT mice

To characterize the specific T cell population activated after arterial injury, we performed flow cytometric analysis on cells in the regional lymph nodes and spleen at various time points after injury. We used previously reported activation markers, namely: CD69, CD28, CD25, and CD44 [10], [21]–[23].

#### CD4+ T cells after arterial injury

**Figure 1:**
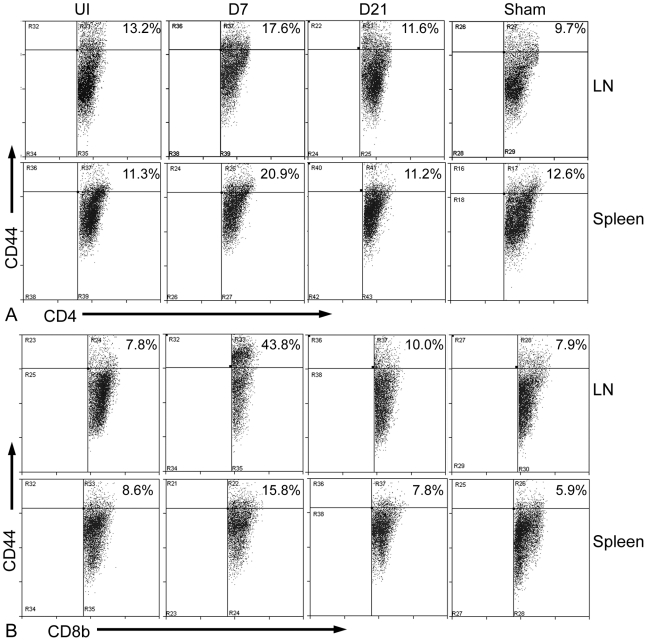
Lymph node and splenic CD44^+^ T cells after arterial injury in WT mice. Representative scatter plots of lymph node (LN) and spleen cells collected at various times after injury and characterized using CD44 gated on CD4 (A) or CD8b (B). Cells were collected from uninjured (UI) mice, or 7 days (D7) and 21 days (D21) after arterial injury. Sham mice correspond to 7 days after sham surgery. Percentage of cells is indicated on the top right corner of each graph.

There was no significant increase in CD4^+^CD69^+^ T cells in the lymph nodes and spleens after injury (not shown), as previously reported [10]. CD4^+^CD44^hi^ T cells in the lymph nodes (Fig. 1A, top panel) and spleen (Fig. 1A, bottom panel) of WT mice significantly increased 7 days after injury. Twenty-one days after injury, CD4^+^CD44^hi^ cells decreased back to uninjured levels (Fig. 1A and Table 1). CD4^+^CD25^+^ and CD4^+^CD28^+^ T cells did not significantly change after injury (Table 1). The sham group did not have significant changes in CD4^+^ T cells (Fig. 1A and Table 1).

**Table 1:** T cell activation after arterial injury.

	UI	D7	D21	Sham
LN CD4^+^CD44^hi^	13.7±3.2	19.1±2.0*	11.5±1.1	12.0±1.4
Spl CD4^+^CD44^hi^	15.5±4.2	22.1±2.1**	11.0±1.7	13.6±1.2
Spl CD4^+^CD25^+^	10.7±4.1	12.6±3.0	10.5±3.5	11.1±0.5
Spl CD4^+^CD28^+^	90.9±2.7	93.0±5.0	93.3±3.4	93.7±0.9
LN CD8b^+^CD44^hi^	9.0±1.0	38.9±13.0**	9.9±0.8	10.6±1.0
Spl CD8b^+^CD44^hi^	8.4±2.7	15.6±3.2*	10.4±2.5	9.6±1.0
Spl CD8b^+^CD25^+^	2.3±0.8	6.7±3.0*	5.9±2.4	2.2±0.5
Spl CD8b^+^CD28^+^	79.3±6.2	91.5±2.3† ‡	90.9±3.3† ‡	83.7±2.3

LN = lymph nodes, N≥3; Spl = spleen, N≥5. UI = Uninjured mice; D7 and D21 = 7 days and 21 days after arterial injury, respectively. All values expressed as percent CD4^+^ or CD8b^+^ gated cells.

*p<0.05 vs. other time points;

**p<0.01 vs. other time points;

† p<0.01 vs. UI;

‡ p<0.05 vs. sham.

#### CD8+ T cells after arterial injury

**Figure 2:**
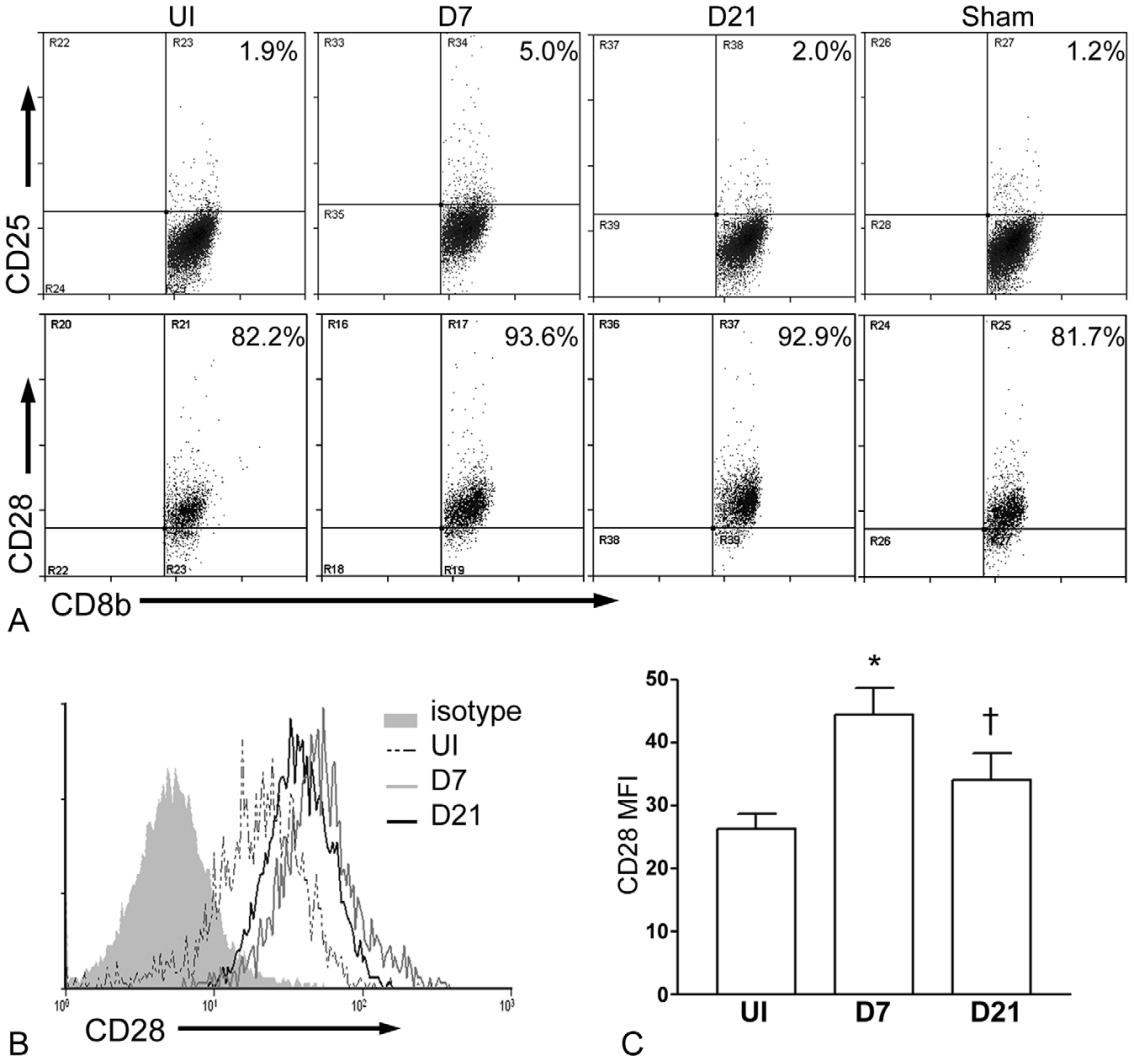
Splenic CD8b^+^ T cells after arterial injury in WT mice. Representative scatter graphs of CD8b-gated CD25^+^ (A, top panel) and CD28^+^ (A, bottom panel) spleen T cells. Representative histogram of CD28 expression on CD8b-gated cells (B). Geometric mean fluorescence intensity (MFI) of CD28 on CD8b-gated cells (C; N = 3–4 each time point).

There was no significant increase in CD8b^+^CD69^+^ T cells in the lymph nodes and spleens after injury (not shown). CD8b^+^CD44^hi^ cells in the lymph nodes (Fig. 1B, top panel) and spleen (Fig. 1B, bottom panel) significantly increased 7 days after injury then decreased 21 days after injury (Fig. 1B and Table 1). Splenic CD8b^+^CD25^+^ T cells also increased significantly 7 days after injury and decreased back to uninjured levels at day 21 (Fig. 2A, top panel and Table 1). CD8b^+^CD28^+^ T cells in the spleen were significantly increased within a week after injury (Fig. 2A, bottom panel and Table 1) and remained elevated 21 days after injury. In addition, CD8b^+^ T cells in the spleen of WT mice had significantly increased CD28 expression 7 days after injury compared with uninjured mice (Fig. 2B and C), suggesting not only increased number but increased CD28 expression as well. There were no significant changes in the percentage of CD8b^+^ T cells in the sham group (Figs. 1 and 2 and Table 1).

#### T cells in the injured arterial wall

**Figure 3:**
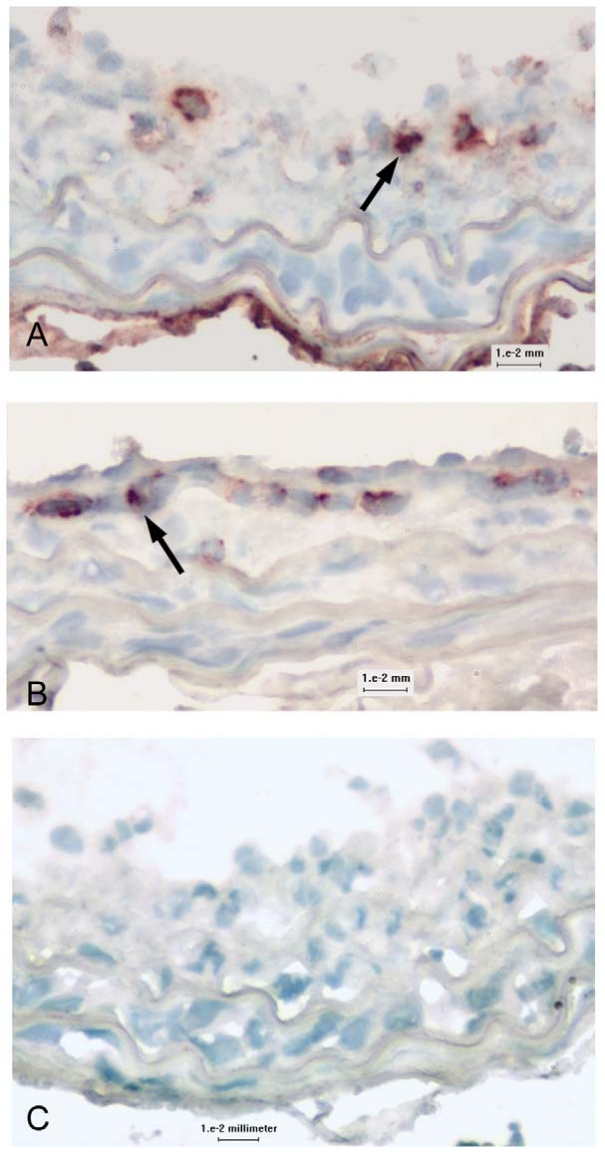
T cells in the injured arterial wall. Representative sections of 21-day injured carotid arteries stained for CD4 (A) or CD8b (B) identify their localization (arrows) in the arterial wall. Omission of primary antibody (C) was used as control for staining. N = 4 each; bar = 10 microns.

Uninjured and 7-day injured arteries had very scant presence of CD4 or CD8b^+^ T cells. CD4^+^ T cells were present in the neointima and advential layers of the carotid artery 21 days after injury (Fig. 3A) with 0.60±0.49% positive stain area (N = 4) of the arterial wall. CD8b^+^ T cells were detected predominantly in the neointima of the injured carotid artery at the same time point (Fig. 3B) with 0.53±0.23% positive stain area (N = 4) of the arterial wall.

### Adoptive cell transfer of CD8^+^ T cells into Rag-1−/− mice reduced neointima formation

**Figure 4:**
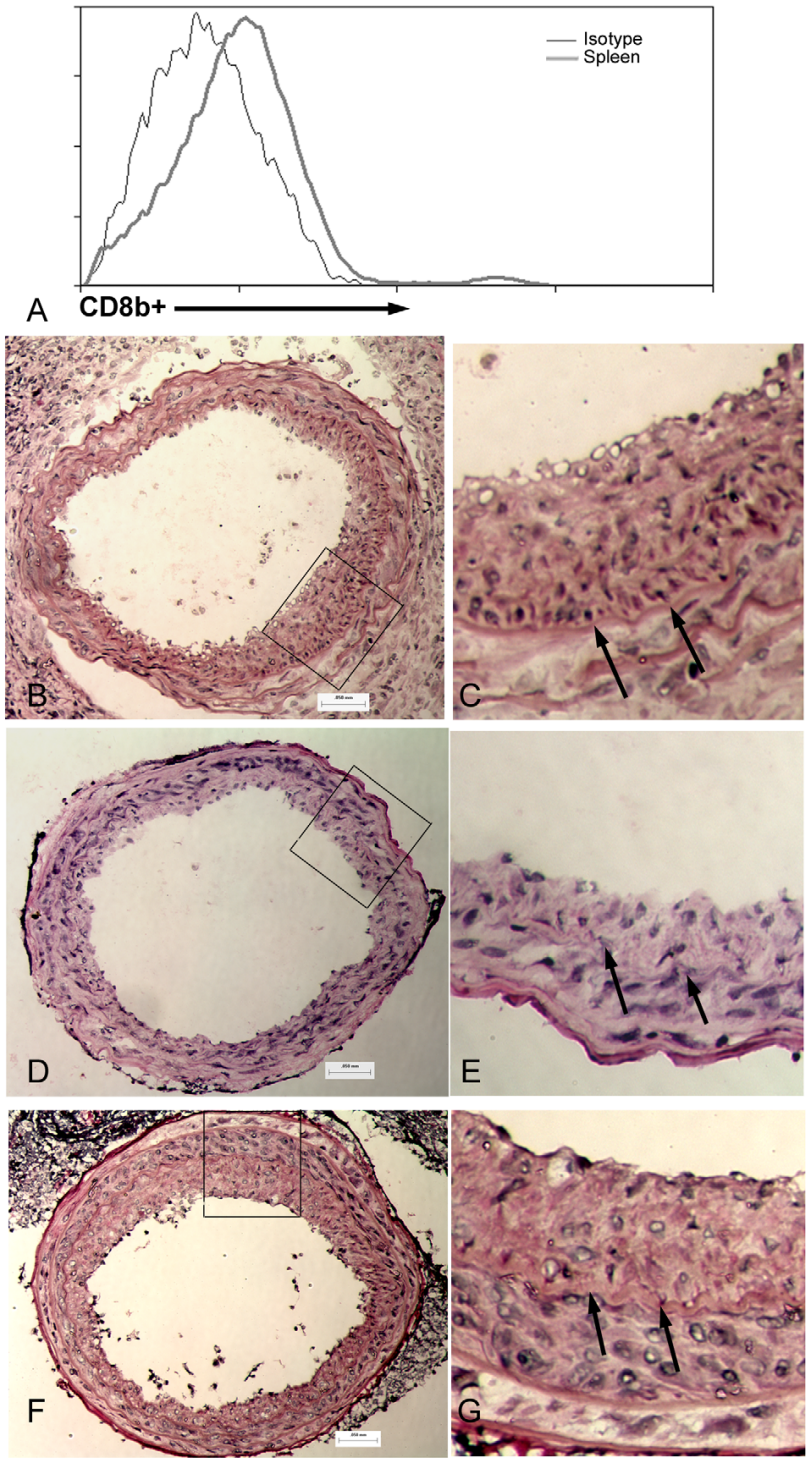
Adoptive T cell transfer. Representative histogram of CD8^+^ T cells homing-into the spleen of a recipient mouse 48 hours after cell transfer, before arterial injury (A). Viable lymphocytes were gated on the FSC/SSC plot. Representative sections of 21-day injured carotid arteries (400× magnification) stained with hematoxylin and eosin from Rag-1−/− mice (B), Rag-1−/− injected with CD8^+^ T cells (D), and Rag-1−/− mice injected with CD4^+^ T cells (F). Boxed area indicates magnification of the respective cross-sections (C, E, G). Arrows indicate internal elastic lamina. Bar = 50 microns.

Injury of WT mice showed that arterial injury itself resulted in T cell activation; we therefore transferred T cells from non-injured donors to assure that the activation signal was from injury of the recipient mice. To determine specificity of the T cell sub type involved, excluding the influence of B cells and immunoglobulin, we performed adoptive transfer of T cells from donor CD4−/− or CD8−/− mice into Rag-1−/− mice. The use of specific T cell knockout models as donors assured the purity and specificity of the T cell population transferred into Rag-1−/− mice, avoiding the reported interaction of CD4 and CD8 T cells during activation. Flow cytometry indicated that CD8b^+^ T cells were present in the spleen of recipient mice 48 hours after adoptive transfer (Fig. 4A), indicating successful cell transfer. Twenty-one days after injury, Rag-1−/− mice that received CD8^+^ T cells (Rag-1+CD8; Fig. 4D and E; Table 2) had significantly reduced neointima area compared with Rag-1−/− mice without cell transfer (Fig. 4B and C, Table 2). Intima-to-media (I/M) ratio was also significantly decreased in Rag-1+CD8 compared with Rag-1−/− mice (Table 2). Rag-1−/− mice that received CD4^+^ T cells (Rag-1+CD4; Figure 4F and G) had similar neointima area and intima∶media ratio (Table 2) compared with Rag-1−/− mice without T cell transfer. There were no significant differences in vessel size as determined by external elastic lamina area (Table 2). We had previously reported that adoptive T cell transfer into Rag-1−/− mice reduced neointima formation after arterial injury [16]. The current results indicate that CD8^+^ T cells are the cell type involved in the T cell inhibition of neointima formation.

**Table 2:** Neointimal thickening 21 days after arterial injury.

	Intimal area (mm^2^)	I/M ratio	EEL (mm^2^)
Rag-1−/− (n = 13)	0.021±0.009	0.54±0.21	0.121±0.025
Rag-1+CD8 T cells (n = 11)	0.011±0.007*	0.27±0.17*	0.106±0.030
Rag-1+CD4 T cells (n = 7)	0.018±0.009	0.40±0.18	0.129±0.009

All values are mean ± SD. Rag+CD8 T cells = Rag-1−/− mice injected with CD8^+^ T cells from CD4−/− donors; Rag-1+CD4 T cells = Rag-1−/− mice injected with CD4^+^ T cells from CD8−/− donors. I/M = intima to media ratio.

*p<0.05 vs. Rag-1−/−.

### Transferred CD8^+^ T cells in the injured artery

**Figure 5:**
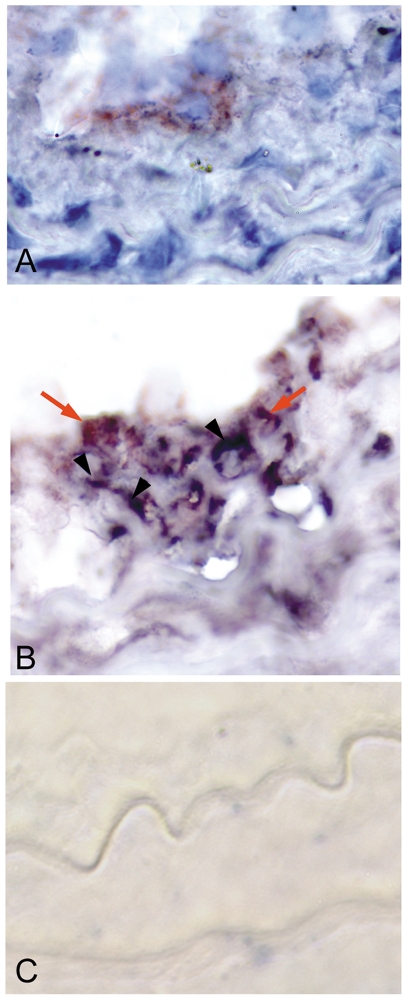
CD8b^+^ cells in the injured artery of recipient Rag-1−/− mice. Detection of CD8b^+^ cells (A; reddish-brown stain) in arteries of recipient Rag-1−/− mice 21 days after injury. Adjacent sections double-stained (B) for CD8b^+^ (orange arrow) and active caspase-3 (dark blue stain, black arrowhead) showed positive cells in close proximity. Omission of primary antibodies was used as control (C). 1000× magnification.

Immuno-histochemical staining showed presence of CD8b^+^ cells in the neointimal layer of Rag-1+CD8 mice (Fig. 5A). Staining of consecutive sections showed presence of CD8b^+^ cells in close proximity to active caspase-3 stain (Fig. 5B) suggesting cytotoxic activity of the CD8b^+^ T cells.

### Cytotoxic activity of CD8^+^ T cells against SMCs

**Figure 6:**
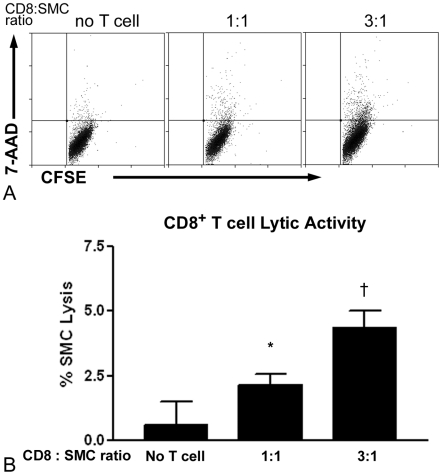
Lytic activity of CD8^+^ T cells from spleens of 21-day injured mice. Syngeneic aortic SMCs were labeled with CFSE and co-cultured for 4 hours with increasing number of CD8^+^ T cells enriched from CD4−/− mouse spleens. SMC lysis was assessed using 7-AAD^+^ cells gated on CFSE (A) and expressed as % SMC lysis (B) relative to basal lysis (see Methods). N = 3–5 each group. *P<0.05 vs. no T cell; †P<0.01 vs. no T cell and 1∶1.

To assess a possible mechanism for CD8^+^ T cell mediated reduction of neointima formation, syngeneic aortic smooth muscle cells (SMCs) were co-cultured for 4 hours in the presence or absence of CD8^+^ T cells from spleens of 21-day injured mice. There was significant cytotoxic activity against SMCs by CD8^+^ T cells at effector to target ratios of 1∶1 and 3∶1 (Fig. 6).

### Characterization of adoptively transferred CD8^+^ T cells

**Figure 7:**
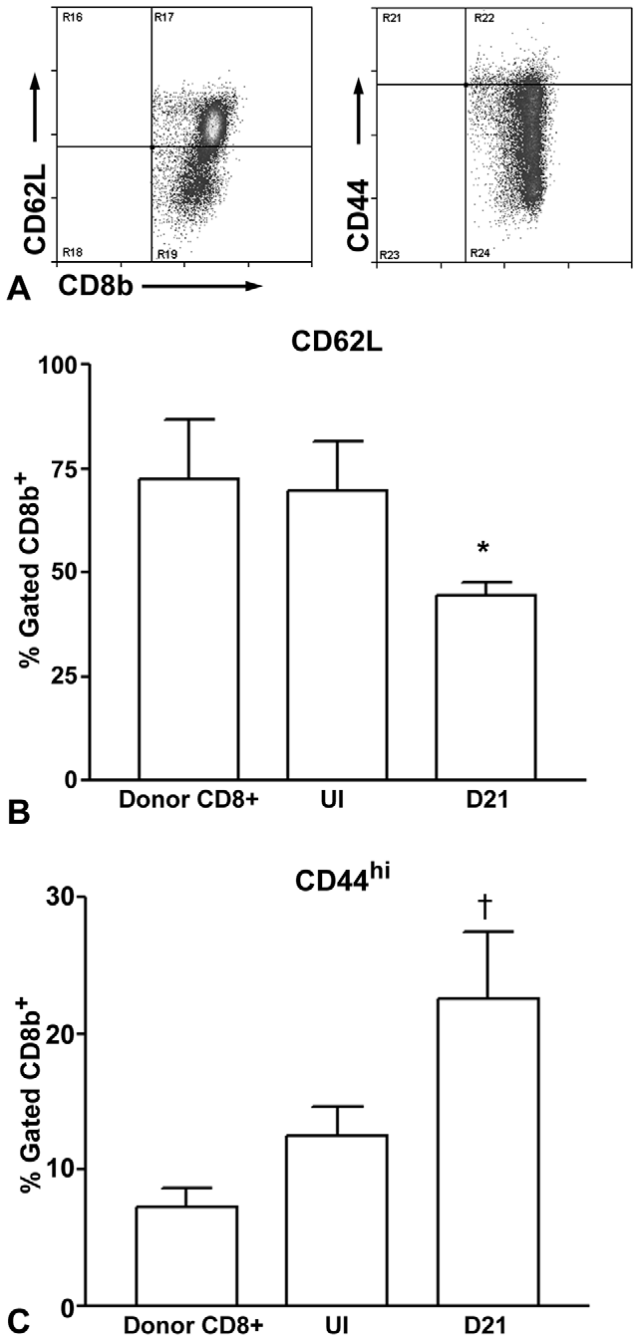
Characterization of cells transferred to recipient Rag-1−/− mice. T cells enriched from pooled spleens of CD4−/− mice (Donor CD8^+^) were used for flow cytometric analysis and compared with T cells enriched from spleens of CD8^+^ T cell recipient Rag-1−/− mice 48 hours after cell transfer without injury (UI), and 21 days after injury (D21). Representative flow cytometric analysis of CD62L or CD44 gated on CD8b^+^ cells from donor mice (A). CD8b^+^CD62L^+^ (B) and CD8b^+^CD44^hi^ (C) cells T cells were compared between Donors (N = 3), UI recipients (N = 4) and D21 recipients (N = 4). *P<0.05 vs Donors and UI; †P<0.01 vs Donors and UI.

Spleen cells were collected from donor mice (designated as Donor CD8^+^), uninjured recipient mice 48 hours after T cell transfer (UI), or recipient mice 21 days after injury (D21). Donor cells were aliquoted from the pooled donor spleens before adoptive transfer. Spleens from 2–3 uninjured recipient mice were pooled due to the small size of spleens, whereas individual spleens were used for the D21 time point. Cells were enriched for T cells and flow cytometric analysis was performed. CD8b^+^CD62L^+^ T cells were unchanged in uninjured recipient mice compared with donor T cells but were significantly reduced 21 days after injury (Fig. 7B). CD8^+^CD44^hi^ T cells increased in uninjured mice and significantly increased further D21 after injury (Fig. 7C).

### Homeostatic T cell expansion in CD8^+^ T cell recipient mice

Although the changes observed in the transferred CD8^+^ T cells suggested activation after injury, homeostatic T cell proliferation also occurred as a consequence of cell transfer, which has been demonstrated in recipients that are immune-deficient [24], [25]. Homeostatic CD8^+^ T cell expansion is dependent on MHC-I signaling [24], [25]. To test if the reduction in neointima formation is dependent on homeostatic CD8^+^ T cell expansion, we used a monoclonal antibody to block MHC-I signaling in the recipient mice. Flow cytometric analysis showed that antibody treatment significantly reduced the percentage of CD8b^+^ T cells in the spleen, suggesting that homeostatic T cell expansion was significantly reduced (Table 3). CD8b^+^CD44^+^ T cells in the spleen were also significantly reduced by antibody treatment but CD8b^+^CD62L^+^ T cells were not significantly different (Table 3). CD8^+^ T cell recipient Rag-1−/− mice treated with MHC-I monoclonal antibody (mAb) or its isotype control still had reduced neointima formation (0.0125±0.004 mm^2^, N = 6 and 0.0142±0.002 mm^2^, N = 5 respectively) compared with Rag-1−/− mice without cell transfer (0.021±0.009 mm^2^, Table 1; p<0.05 vs. mAb treatment). The reduction in neointima formation was to a similar extent as untreated CD8^+^ T cell recipient mice (see Table 2). The results showed that even as MHC-I antibody treatment significantly reduced homeostatic CD8^+^ T cell expansion in the recipient mice, inhibition of neointima formation by CD8^+^ T cells was not significantly affected.

**Table 3:** Effect of MHC-I mAb treatment on CD8^+^ T cells of recipient mouse spleens.

	MHC-I mAb	IgG isotype
CD8b**^+^** Splenocytes	20.3±4.6%*	31.7±5.3%
CD8b^+^CD62L^+^ Splenocytes	15.8±2.1%	14.1±2.4%
CD8b^+^CD44^hi^ Splenocytes	10.2±2.9%*	14.5±2.8%

Analysis was performed on CD8b gated lymphocytes. All values are mean ± SD. MHC-I mAb (monoclonal antibody) N = 6; IgG isotype N = 5;

*p<0.05.

### CD8^+^ T cells from WT donors also reduced neointima formation

CD8^+^ T cells from CD4−/− mice have been reported to be contaminated by an atypical MHC-II restricted population [26]. To ascertain whether the reduction in neointima formation by adoptive cell transfer of CD8^+^ T cells into Rag-1−/− mice was due to this atypical population from CD4−/− mice, we used WT mice as donors instead. WT CD8^+^ T cell transfer significantly reduced neointima area in recipient Rag-1−/− mice compared with Rag-1−/− mice without cell transfer (0.014±0.008, N = 12 vs. 0.021±0.009 mm^2^, N = 13, respectively; P<0.05). Intima∶media ratio was also significantly reduced by WT CD8^+^ T cell transfer compared with Rag-1−/− mice without cell transfer (0.32±0.15 vs. 0.54±0.21, respectively; P<0.01). The results further support the finding that CD8^+^ T cells reduce neointima formation.

## Discussion

The results of our study provide the following key findings: 1) T cells are activated and respond to arterial injury; 2) CD8^+^ T cells have a more robust and sustained response; 3) adoptive transfer of T cell subtypes identify specific CD8^+^ T cell function in response to arterial injury; and 4) the effector function of CD8^+^ T cells in arterial injury involves the cytotoxic response. In addition, we also provide supporting evidence that the specific role of CD8^+^ T cells in our adoptive cell transfer studies is not dependent on homeostatic proliferation, or on an atypical CD8^+^ T cell population from the CD4−/− donor mice.

There is currently a lack of studies that define T cell response to injury. This is highlighted by the current dilemma in the use of drug-eluting stents coated with immune-suppressants to treat restenosis post PCI. The treatment has been suggested to result in incomplete healing of the injured artery [4], [5], a process which remains incompletely understood, suggesting that the immune system regulates vascular tissue homeostasis after injury [6]. Previous reports indicate that T cells inhibit neointima formation after arterial injury [14], [16], [18], although conflicting results have been reported [10]–[13]. These findings underscore the need to define the T cell response to arterial injury. We did not observe an increase in CD69^+^ T lymphocytes after arterial injury, similar to another report [10]. However, flow cytometry using CD44, CD28, and CD25 suggested that both CD4 and CD8 T cell activation occurs after arterial injury in the WT mice. The activation phenotypes observed appears to be the specific result of injury to the carotid artery since the pattern of activation was not observed in the sham group.

Previous characterization of our injury model showed the endothelial lining of the intima sloughing off within a week [20]. Capture and processing of apoptotic and necrotic cells occur in the lymph nodes and spleen through phagocytosis by dendritic cells and macrophages which then signal either immune activation or tolerance [27]. Thus, presentation of, and activation in response to self-antigens would predictably occur at these sites and our results support this notion. Our results further suggest that CD4^+^ T cell activation was rather transient, yet CD8^+^ T cell activation was robust and sustained. Immuno-histochemistry identified the local presence of both T cell subtypes in the carotid arteries 21 days after injury, supporting the previously reported involvement of T cells in inhibiting neointima formation [14], [16], [18].

We assessed the specific role of sub-populations of T cells using immune deficient Rag-1−/− mice as recipients of adoptive T cell transfer and show that CD8^+^ T cells are the primary T cell type involved in reducing neointima formation. Several reports indicate that immune deficiency in the Rag−/− mouse model results in augmented neointima formation after arterial injury [15]–[17], [20]. The adoptive transfer approach we used was designed to show that any effect of a T cell sub-type in a combined immune-deficient recipient such as the Rag-1−/− mouse would be specific to that T cell sub-type since Rag-1−/− mice have no mature T cells and B cells. The presence of B cells or immunoglobulin would complicate the interpretation of the role of specific T cell types given our previous report on the role of B cells and natural antibodies in neointima formation [15], [20]. Separate experiments need to be designed to address the possible role of the interaction between specific T cell subsets and B cells in the setting of arterial injury and neointima formation. The results of the adoptive transfer experiments show that CD8^+^ T cells are directly and specifically involved in limiting neointima formation. To our knowledge, this is the first study to show direct evidence of this specificity. It is also clear from our studies that specific arms of the immune system differentially respond to, and affect neointima formation [15], [16], [20].

Our results suggest that cytotoxic activity against SMCs is at least one mechanism involved in the CD8^+^ T cell response after injury. Immuno-histochemical staining of arteries of recipient Rag-1−/− mice shows CD8b^+^ cells in close proximity to active caspase-3 positive cells, suggesting cytotoxic activity. The *in vitro* co-culture experiment supports the cytotoxic pathway of control of SMCs by CD8^+^ T cells in the injured arterial wall. However, it is also worth noting that syngeneic SMC lysis by CD8^+^ T cells was not in the magnitude of what is observed in an allogeneic response, suggesting that only a small number of SMCs are targeted by CD8^+^ T cells.

Our results also show that the process of adoptive transfer by itself results in CD8^+^ T cell phenotypic change, evidenced by increased CD8^+^CD44^hi^ cells. This has been attributed to homeostatic expansion of the transferred cells [24], [25]. The lack of change in CD8^+^CD62L^+^ cells by adoptive transfer by itself is in agreement with the reported effect of homeostatic cell expansion on cell phenotype [25]. There appears to be either further immune activation as a result of arterial injury, or continuous homeostatic cell expansion, since CD8^+^CD44^hi^ T cells increased further and CD8^+^CD62L^+^ cells decreased at the day 21 time point.

The observation that phenotype change associated with homeostatic CD8^+^ T cell expansion had occurred led us to test if the effect on neointima formation was directly due to this process. Homeostatic T cell expansion after adoptive transfer into immune-deficient recipients is MHC-I dependent [24], [25]. Treatment of the recipient mice with an anti-MHC-I monoclonal antibody partially suppressed homeostatic expansion, reducing the CD8^+^CD44^+^ T cells by about half yet still reducing neointima formation, suggesting that the effects of transferred CD8^+^ T cells on neointima formation are at least partially independent of homeostatic expansion.

The transfer experiments also suggest that CD4^+^ T cells have no direct role in modulating neointima formation. Although the result is unexpected given the pathogenic role of CD4^+^ T cells in lipid-rich atherosclerotic plaques [28], there is yet to be a definitive report on its role in arterial injury. There is the predominant yet untested speculation that CD4^+^ T cells are directly involved in neointima formation. Reduced neointima formation by CD4 antibody treatment in the rat injury model was attributed to effects on macrophage [29]. Vasculitogenic T cells, predominantly CD4^+^ that are activated and respond to syngeneic SMCs, have been previously studied. However, these cells were first primed by in vitro exposure to SMCs and then transferred into recipients and shown to cause vasculitogenic injury [30]. This study is unlike our report where the T cell response is characterized after arterial injury in vivo. Furthermore, we transferred naïve, non-primed T cell subtypes into immune-deficient recipients to assure that the T cell activation signal was from the injury of the recipients' carotid artery. Thus, our results are the first to directly assess the role of CD4^+^ T cells in arterial injury and neointima formation.

CD4−/− and CD8−/− mice were chosen as donors in the study to assure the purity and specificity of the T cell population transferred into Rag-1−/− mice. This is significant given the known interaction between CD4 and CD8 T cells during activation and response. Although the results strongly support a significant role for CD8^+^ T cells in inhibiting neointima formation after injury, interpretation must be made with caution in the context of the reported contamination of MHC-II restricted CD8 T cells in CD4−/− mice [26]. However, additional experiments using WT mice as donors confirm the finding that CD8^+^ T cells reduce neointima formation and that the effect of the adoptive transfer of T cells from CD4−/− mice is not due to the atypical cell population.

The specific signaling pathways involved in T cell activation after arterial injury remain to be delineated. However, it has been reported that mice with deficiency in hypoxia-inducible factor 1α (Hif-1α) specifically in T cells had increased neointima formation in response to arterial injury [13]. Hif-1 activity is increased in cells subjected to hypoxic conditions [31]. However, Hif-1 activation occurs through both hypoxic and non-hypoxic pathways [32], [33], both of which occur after arterial injury. Activation through T cell receptor signaling increases Hif-1α expression in T cells [34] and T cell function is regulated by Hif-1α [35]. Hypoxia and inflammation interact in both innate and adaptive immune responses [36] and it is unclear how this interaction factors in neointima formation after arterial injury. For example, it is unknown how dendritic cells respond to hypoxia in our injury model. What may link the two arms of the immune system in the context of hypoxia and inflammation is signaling through the Toll-like receptors. Hif-1α mediates TLR2 and TLR6 expression [37]. How this relates to arterial injury remains uncertain because of conflicting reports on the role of endogenous TLR2 signaling after arterial injury [38], [39] and TLR6 signaling after arterial injury remains to be defined. However, the reported interaction between TLR2 and TLR6 [37] could yet link Hif-1α activation, TLR signaling, and the immune response to arterial injury.

The limitation of the study is inherent in the model used. The injury performed is on arteries that are neither diseased nor have on-going inflammation, thus not completely replicating human stenotic vessels. However, our study shows that CD8^+^ T cells control SMC accumulation in the neointima layer of the injured artery, which in the simple case of narrowing of the artery is beneficial. How this activity of CD8^+^ T cells may influence an advanced atherosclerotic plaque remains unknown. It also remains unclear what specific activation signal would result in a cytotoxic response of CD8^+^ T cells against vascular SMC in the arterial intima, and what makes a relatively small number of SMCs susceptible to targeting by CD8^+^ T cells. One possibility is the notion of the “altered-self” phenotype of injured SMCs, which could turn them into target cells of immune surveillance. Altered-self recognition is an essential aspect of homeostasis [27].

In conclusion, the report identifies CD8^+^ T cells as the specific and selective T cell type that inhibits neointima formation after arterial injury. There is a surprising lack of direct effect of CD4^+^ T cells in the response to arterial injury in the absence of interaction with either B cells or CD8^+^ T cells. The findings implicate CD8^+^ T cells in regulating neointima formation and warrants further studies of the process that defines this effect.

## Materials and Methods

### Arterial injury

Mice were housed in a pathogen-free facility and had ad libitum access to food and water. Aseptic peri-adventitial cuff injury was performed on the right carotid artery of 25 week old male wild type (WT) or Rag-1−/− mice on the C57Bl6/J background (Jackson Laboratory), as previously described [40]. Briefly, mice were anesthetized with ketamine and xylazine and carprofen was administered prior to surgery for post-surgical pain relief. The right carotid artery was dissected and exposed, and a 2.5 mm-long Tygon tube (internal diameter of 0.51 mm) with a longitudinal opening was placed around the right carotid artery, secured with ligatures around it, and the wound was closed with sutures. A prior report detailed the injury with an electron microscopic study showing that the procedure results in the endothelial layer sloughing off within a week [20]. Uninjured mice served as control. An additional control group of WT mice had sham injury where surgical manipulation of the mice was performed without peri-adventitial cuff injury. Carotid arteries were harvested after perfusion with normal saline for 10 minutes. The Cedars-Sinai Institutional Animal Care and Use Committee approved the experimental protocols specifically used in this study (IACUC # 001552 and 002868).

### Morphometric Analysis

Frozen sections 6–8 µm thick were collected from the injured carotid arteries of Rag-1−/− mice, stained with hematoxylin and eosin, and the vessel area measured as described previously using image analysis software (ImagePro) [16].

### Flow cytometry

Regional lymph nodes and the spleen were collected and subjected to red blood cell lysis. Cells were then stained with CD4 or CD8b and CD69, CD44, CD25, or CD28 (BD Bioscience) for flow cytometry.

### T cell enrichment and transfer

Spleen cells from age-matched, donor CD8−/− or CD4−/− mice were pooled. The cells were then negatively selected for T cells using a commercially available kit (Invitrogen) with paramagnetic beads and a magnetic particle concentrator, as recommended by the manufacturer (Dynal). Flow cytometry confirmed cell enrichment with 90.1±2.3% T cells. Equal numbers of cells from either CD4−/− or CD8−/− donors were then injected via the tail vein of Rag-1−/− mice (3×10^7^ cells/mouse) 48 hours prior to injury [16]. Age-matched WT mice were also used as CD8^+^ T cell donors to confirm results from the CD4−/− donors. Cells were collected from lung, lymph node and spleen for flow cytometry 48 hours after cell transfer to assess homing. At least two separate isolation and transfer procedures were performed for each group subjected to arterial injury.

### Characterization of transferred cells

T cells enriched from CD4−/− donor mouse spleens and T cells enriched from recipient spleens were collected and double-stained for CD8b and CD62L, or CD44 for flow cytometric analysis. Matching isotype antibodies were used as control.

### MHC-I antibody treatment

MHC-I antibody treatment in CD8^+^ T cell recipient Rag-1−/− mice was performed using a mouse monoclonal anti-H-2D^b^ antibody (BD Biosciences; 0.2 mg/mouse, i.p.) on the day of cell transfer, at injury, and twice weekly until day 14. Mouse IgG isotype treatment was used as control. Mice were euthanized 21 days after injury.

### Immunohistochemistry

Frozen sections 6–8 µm thick were fixed in ice-cold acetone and stained for CD8b (BD Bioscience). Biotin-conjugated secondary antibody was used for detection and visualized using horseradish peroxidase-conjugated streptavidin and AEC (DAKO). Biotin-conjugated anti-active caspase-3 (BD Biosciences) was used to double stain sections for CD8b and active caspase-3. Detection was performed using alkaline phosphatase-conjugated streptavidin and BCIP/NBT (DAKO). Omission of the primary antibodies was used as negative control.

### SMC lytic assay

An aliquot of CD8^+^ T cells negatively purified from 21-day injured CD4−/− mice were co-cultured with CFSE-labeled (1.0 µM) syngeneic aortic smooth muscle cells (SMC) in 10% FBS/DMEM for 4 hours in 37°C at a CD8^+^ T cell to SMC ratio of 1∶1 or 3∶1. Basal lysis of SMC without T cells was used as control. Cells were washed in 1× PBS, trypsinized to detach the SMC monolayer, and stained with 7-AAD for cell lysis as previously described [41]. Flow cytometry was performed to detect 7-AAD-stained viable SMCs gated on FL1 (CFSE^+^). Gating on CFSE assured viability of the cells that were analyzed [41]. Results are presented as % SMC lysis:
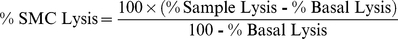
where sample lysis is SMC lysis in the presence of CD8^+^ T cells at a given effector∶target ratio; and basal lysis is SMC lysis without CD8^+^ T cells [41].

### Statistics

Results are presented as mean ± SD. ANOVA was used to test for significance followed by Neuman-Keuls post test, unless indicated otherwise. Significance was set at p<0.05.
